# Sex‐Specific Variation in Foraging Behavior is Related to Telomere Length in a Long‐Lived Seabird

**DOI:** 10.1002/ece3.70732

**Published:** 2024-12-17

**Authors:** Mauricio Guillen‐Parra, Rocio Barcenas‐Flores, Alberto Velando, Anne Wiley, Bibiana Montoya, Roxana Torres

**Affiliations:** ^1^ Programa de Posgrado en Ciencias Biológicas Universidad Nacional Autónoma de México Ciudad de México Mexico; ^2^ Departamento de Ecología Evolutiva, Instituto de Ecología Universidad Nacional Autónoma de México Ciudad de México Mexico; ^3^ Animal Ecology Group (GEA), Centro de Investigacion Mariña (CIM) Universidade de Vigo Vigo Spain; ^4^ Department of Natural Sciences Bowie State University Bowie Maryland USA; ^5^ Estación Científica La Malinche, Centro Tlaxcala de Biología de la Conducta (CTBC) Universidad Autónoma de Tlaxcala Tlaxcala Mexico

**Keywords:** chick growth rate, chick provisioning rate, foraging behavior, long‐lived seabird, telomere length

## Abstract

Foraging during breeding is a demanding activity linked to breeding investment and possibly constrained by individual quality. Telomere length, the protective nucleoproteins located at the ends of the chromosomes, is considered a trait reflecting somatic maintenance and individual quality. Therefore, foraging effort and parental investment may be positively related to telomere length, if individuals with longer telomeres are of better quality and thus able to maintain better body condition and allocate more resources to parental activities. In the brown booby ( *Sula leucogaster* ), we investigated if telomere length is related to body mass (a proxy of condition) and whether variation in foraging behavior and provisioning effort is related to telomere length or body mass. Then, we explored whether variation in foraging and provisioning influences the chick mass growth rate. In 34 pairs nesting in Isla de San Jorge, in the Gulf of California, México, we sampled their blood to estimate telomere length, measured their body mass, and for 10 days, recorded their foraging behavior via global positioning system (GPS) loggers and their chick provisioning rate and chicks' mass growth rate. We found a positive relationship between parents' body mass and telomere length. Body mass did not affect foraging behavior. Females with longer telomeres were more prone to travel longer distances toward offshore and deeper waters than females with shorter telomeres. In contrast, males with longer telomere lengths performed more nearshore foraging trips than males with shorter telomeres. The chick provisioning rate was unrelated to telomere length or body mass, but females fed the chick at a rate 2.4 times greater than males. Females' offshore foraging, but not males', was positively related to chick mass growth rate. Our results suggest that individual quality, indicated by telomere length, is an important driver of sex‐specific, between‐individual variation in foraging behavior, indirectly affecting offspring condition.

## Graphical abstract

Foraging during breeding is energetically demanding, intimately linked to breeding investment, and possibly constrained by individual condition. Telomere length, the protective nucleoproteins located at the ends of the chromosomes, is considered a trait reflecting somatic maintenance and individual quality. We found that brown booby females with longer and average telomeres (presumably in better condition) were more prone to travel longer distances toward more offshore and deeper waters than females with shorter telomeres (presumably in lower condition), while males exhibited the opposite foraging pattern suggesting that individual condition is an important driver of sex‐specific, between‐individual variation in foraging behavior.

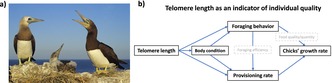

## Resumen

El forrajeo durante la reproducción es una actividad demandante ligada a la inversión reproductiva y posiblemente limitada por la calidad individual. La longitud de los telómeros, nucleoproteínas protectoras ubicadas en los extremos de los cromosomas, se considera un rasgo que refleja el mantenimiento somático y la calidad individual. Por lo tanto se espera que el esfuerzo de forrajeo y la inversión parental esten relacionados positivamente con la longitud de los telómeros, si los individuos con telómeros más largos son de mejor calidad y sean capaces de mantener una mejor condición corporal y asignar más recursos a las actividades parentales. En el bobo café ( *Sula leucogaster* ), investigamos si en los padres la longitud de los telómeros está relacionada con su masa corporal (un indicador de la condición) y si la variación en el forrajeo y y el esfuerzo en alimentaciones está relacionada con la longitud de los telómeros o la masa corporal. Luego, exploramos si la variación en el forrajeo y la tasa de alimentaciones influye en la tasa de crecimiento en masa de las crías. En 34 parejas que anidaban en la Isla de San Jorge, en el Golfo de California, México, tomamos muestras de su sangre para estimar la longitud de los telómeros, medimos su masa corporal y, durante 10 días, registramos su comportamiento de forrajeo, a través de registros de sistemas de posicionamiento global (GPS), y su tasa de alimentación a las crías, así como la tasa de crecimiento de las crías. Encontramos una relación positiva entre la masa corporal de los padres y la longitud de los telómeros. La masa corporal no afectó el comportamiento de forrajeo. Las hembras con telómeros más largos fueron más propensas a viajar distancias más largas y distantes de la costa en aguas más profundas que las hembras con telómeros más cortos. Por el contrario, los machos con longitudes de telómeros más largas realizaron más viajes de forrajeo cerca de la costa que los machos con telómeros más cortos. La tasa de alimentación no se relacionó con la longitud de los telómeros o la masa corporal, pero las hembras alimentaron a las crías a una tasa 2.4 veces mayor que los machos. El forrajeo de las hembras, pero no el de los machos, se relacionó positivamente con la tasa de crecimiento de las crías. Nuestros resultados sugieren que la calidad individual, indicada por la longitud de los telómeros, es un factor importante en la variación entre individuos en el comportamiento de forrajeo, lo que afecta indirectamente la condición de la descendencia.

## 1 Introduction

Foraging is an energetically demanding activity crucial for survival and reproduction that imposes costs on individuals (Shaffer, Costa, and Weimerskirch 2003; Mullers et al. 2009). Foraging can be particularly challenging during reproduction, as individuals must obtain enough food to meet their nutritional needs and those of their offspring. Furthermore, while caring for their young, parents may be constrained in foraging time and range by offspring's specific needs, such as optimal food for growth and time required for chick attendance (Charrassin et al. 1998; Shaffer, Costa, and Weimerskirch 2003; Thaxter et al. 2009). Thus, a trade‐off between foraging for self‐maintenance and parental care is expected. The way individuals balance this trade‐off may affect their breeding investment, including the frequency of chick provisioning and the amount and quality of food provided to their offspring (Mariette et al. 2011; Saraux et al. 2011; Jacobs, Elliott, and Gaston 2013), and consequently impact offspring growth and survival prospects (Jakubas et al. 2013). Theoretical and empirical evidence indicates that individual quality can have a strong role in how individuals balance their allocation strategy between foraging for self‐maintenance and parental care (McNamara and Houston 1996; Weimerskirch 1998; Lescroël et al. 2020). Interestingly, sexual differences in foraging and breeding strategies are widespread across animal taxa (e.g., mammals: Lonstein and De Vries 2000; Beck et al. 2003; Kienle et al. 2022; birds: Gow and Stutchbury 2013; Camphuysen et al. 2015; Phillips et al. 2017; fish: Magurran and Garcia 2000), which may lead to sex‐specific, optimal resolution of trade‐offs between foraging for self‐maintenance and reproduction. Thus, individual quality may underlie between‐individual and sex‐specific variation in the allocation to foraging and parental effort (Weimerskirch 1998; Lescroël et al. 2020).

Telomere length has been proposed as an integral indicator of individual quality (Angelier et al. 2019). Telomeres are nucleoproteins located at the ends of the chromosomes (Blackburn 1991). Telomeres maintain cellular integrity by protecting the DNA from incomplete‐replication errors and preventing chromosome‐to‐chromosome fusions (Blackburn 1991; Campisi et al. 2001). However, during cell division, telomeres shorten leading to cellular senescence or apoptosis when reaching a critical length (Campisi et al. 2001). In vertebrates, telomere length at birth is highly dependent on genetic inheritance (Chik et al. 2022); thereafter, the rate of telomere attrition depends on various factors, including the amount of stress an individual experiences and the extent of investment in demanding activities such as growth and reproduction throughout their lives (Eppel et al. 2004; Mizutani et al. 2013; Angelier et al. 2018; Heidinger et al. 2012; Noguera and Velando 2019; Bae et al. 2021). Telomere length of vertebrates negatively correlates with mortality risk (Wilbourn et al. 2018), and in some bird species, telomere length during early development positively correlates with individual lifespan and total reproductive success (Heidinger et al. 2012; Eastwood et al. 2018). Hence, telomere length may function as a biomarker positively reflecting individual quality (Bauch, Becker, and Verhulst 2013; Le Vaillant et al. 2015; Angelier et al. 2019), which is ultimately related to traits like parental care (Viblanc et al. 2020).

Longer telomeres are expected to be associated with increased foraging effort and breeding investment due to their positive relationship with individual quality. However, studies evaluating this correlation in birds have yielded mixed results (Angelier et al. 2019; Sudyka 2019). In some species, individuals with longer telomeres have earlier laying dates, higher provisioning rates, and higher offspring survival (Le Vaillant et al. 2015; Criscuolo et al. 2018; Angelier et al. 2019; Benowitz‐Fredericks et al. 2022); however, opposite patterns have also been observed (Bauch, Becker, and Verhulst 2013; Bauer et al. 2018). The relationship of foraging with telomere length in breeding birds has been scarcely studied. For example, in the thick‐billed murres ( *Uria lomvia* ), there was no relationship between telomere length and foraging distance or the number of trips traveled; however, individuals with shorter telomeres spent less time foraging underwater and dived deeper than individuals with longer telomeres, suggesting that murres with shorter telomeres forage efficiently (Young et al. 2015; Young et al. 2016). Supporting the idea that telomere length is an indicator of individual quality, black‐browed albatrosses ( *Thalassarche melanophrys* ) with longer telomeres performed shorter foraging trips (associated with higher chick provisioning) and produced a greater number of offspring during the 8 years after foraging was recorded (Angelier et al. 2019). In black‐legged kittiwakes ( *Rissa tridactyla* ), males with longer telomeres foraged further from their colonies and fledged more offsprings than males with shorter telomeres (Benowitz‐Fredericks et al. 2022). Finally, in size dimorphic Andean condors ( *Vultur gryphus* ), males traveling longer distances from their nests to their foraging grounds exhibited longer telomeres, while the opposite pattern was found in females, resulting in a pattern of sex‐specific spatial structure related to telomere length (Gangoso et al. 2016). Nevertheless, telomere length was not associated with Andean condors' estimated home range size (Gangoso et al. 2016). Thus, few studies have investigated the relationship between telomere length and foraging behavior yielding mixed results, and seldom sexual differences in this relationship have been investigated.

**FIGURE 1:**
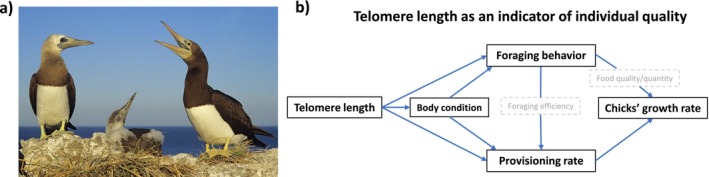
(a) Brown booby family. Photo taken by Claudio Contreras Koob. (b) Hypothesis and predictions tested in this study. We hypothesized that telomere length indicates individual quality and predicted that telomere length may be linked to individuals' body mass (a proxy of body condition), foraging behavior, and provisioning rate. Individuals' condition may further influence foraging behavior and provisioning rate. We expected that variation in foraging behavior may influence the growth rate of chicks directly, for example, through differences in the quality or quantity of food provided or indirectly by affecting the provisioning rate due to differences in foraging efficiency. Finally, the growth rate of chicks may also be directly influenced by the parents' provisioning rate. Gray boxes show traits not evaluated in this study.

In the brown booby ( *Sula leucogaster* ), we investigated if telomere length, as an indicator of individual quality, underlies interindividual and sex‐specific variation in foraging behavior and provisioning rate of breeding adults, ultimately impacting the chicks' growth rate. The brown booby is a pantropical distributed long‐lived seabird, with a maximum lifespan of up to 30 years (Nelson 1978; Yap et al. 2021). Brown boobies display reversed sexual size dimorphism (Nelson 1978). In our study colony at Isla de San Jorge, females are, on average, 25% heavier than males. Brown boobies are socially monogamous with a long period of biparental care. Both parents incubate on average for 42 days a clutch that varies from 1 to 2 eggs and feed the chicks (generally only one chick as they show obligate siblicide) through direct mouth‐to‐mouth food transfer for around 3–4 months before the offspring reach independence (Nelson 1978; Montoya and Torres 2015). Throughout their range, brown boobies breed under variable environmental conditions and forage in open ocean and coastal waters, depending on food availability (Nelson 1978; Schreiber and Norton 2002). During breeding, males and females often differ in their foraging behavior. Females tend to have longer foraging trips in time and distance compared to males in some breeding colonies (Gilardi 1992; Weimerskirch et al. 2009; Miller et al. 2018; Correia et al. 2021), but the opposite trend (Lewis et al. 2005), or no differences between sexes has been reported in other colonies (Castillo‐Guerrero et al. 2016). At Isla de San Jorge, no overall sexual differences in foraging trip duration or distance have been reported, yet on average females reach greater depths than males when diving (Castillo‐Guerrero et al. 2016). In the brown booby, skin color, especially males' gular color, is a sexual trait reflecting direct (i.e., provisioning effort) and indirect (i.e., chick's intrinsic condition) fitness benefits (Montoya and Torres 2015). Interestingly, gular color is repeatable through time and related to individual foraging habits and diet (Michael et al. 2018; Montoya, Flores, and Torres 2018). Furthermore, at Isla de San Jorge, it was recently found that telomere length is positively mirrored in the gular and feet skin color of males and females, respectively; males with longer telomeres had greener gulars, whereas females with longer telomeres had yellower feet (Guillen‐Parra et al. 2024). Brown booby males that forage at more offshore locations and depend on more pelagic diets, both before breeding and during the courtship period, display more sexually attractive skin color during courtship compared to males that forage at nearshore locations and rely on more benthic diets (Michael et al. 2018). These relationships suggest that offshore foraging patterns are related to a better individual condition (Michael et al. 2018); although, the particular foraging variables associated with individuals in better condition may differ between colonies, reflecting locally adapted optimal foraging (Michael et al. 2024). Hence, if telomere length is a marker that reflects the individual quality (Angelier et al. 2019) and ultimately their capacity for parental effort (Viblanc et al. 2020), we predicted that telomere length would be linked to body mass (here, considered as a proxy of individual condition; Labocha and Hayes 2012), foraging behavior and chick provisioning rate, which would impact the chicks' growth rate (Figure 1). Specifically, we predicted that compared to individuals with shorter telomeres, individuals with longer telomeres would have greater body mass, more offshore foraging trips, higher chick provisioning rates, and consequently, their chicks would have a higher mass growth rate. As males and females may differ in their optimal resolution of trade‐offs between foraging for self‐maintenance and reproduction, we tested for differences between sexes in the relationship of individual telomere length with foraging behavior and provisioning rate.

## 2 Methods

The study was carried out from December 2017 to January 2018 in the brown booby breeding colony of Reserva Federal Isla de San Jorge, Sonora Mexico. A total of 34 pairs rearing only one chick were marked with a numbered flag, and both parents were banded (Interrex, Poland) and their mass (±20 g) and the length (±1 mm) of their ulna were measured. From both adults in the nest, we obtained a blood sample (1.5 mL) from their brachial vein using a sterile and heparinized syringe. Then, for a subset of pairs (*n* = 27) foraging behavior was recorded by setting both members of a pair with a global positioning system (GPS) logger for roughly 10 days. To evaluate the potential effects of carrying a GPS logger on parental behavior, the seven remaining focal pairs were treated identically to the birds with GPS, except they were not given a logger.

We captured all chicks from focal nests to obtain a blood sample (1 mL) and measured their beak (±0.1 mm), ulna (±1 mm), and mass (± 1 g), once when their parents were initially captured and again 10 days later, to estimate mass increase during the same period when the parents were GPS tracked. Chicks' morphological measures at first capture were used to calculate their age (mean age = 26, range = 20–32 days) using growth curves from brown booby chicks of known age previously recorded by our group. We molecularly sexed the chicks through DNA extractions from the red blood cells and PCR amplification (Fridolfsson and Ellegren 1999). We performed behavioral observations during the 10 days when the birds were GPS tracked (details below). All blood samples were kept cold until they were centrifuged within 2 hours from collection at 10,000 rpm for 10 min to separate the plasma from the red blood cells, which were then conserved in absolute ethanol and refrigerated at 4°C for either one or 4 years until laboratory analysis, as samples were analyzed in two separate batches (details in section Telomere length).

### 2.1 Foraging Behavior

To measure foraging behavior, we attached a portable GPS logger (i‐gotU GT‐100, MobileAction Technology) to the underside of three to four central rectrices using waterproof adhesive tape (Tesa 4651, Norderstedt, Germany). Before deployment, GPS units were taken out of their original cases and encapsulated in a shrink‐waterproof plastic tube. The weight of the GPS units was 20 g, which represents 1.7% and 2.2% of the total weight of females and males, respectively. After an average, 10.56 ± 1.19 days (range = 7–14), we removed the GPS loggers from all individuals.

The GPS loggers were programmed to register latitude, longitude, speed, time, and date at 3‐min sampling intervals. We defined foraging trips as a bird traveling, only during the day, for at least 30 min at a distance of at least 1.5 km away from the colony, and we considered the trips as concluded when the bird returned to the colony (Michael et al. 2018; Michael et al. 2024). Data were downloaded from the GPS units and analyzed using the software @tripPC. We only analyzed individuals who completed a minimum of three foraging trips during the 10 days of foraging recordings (only one individual with one foraging trip was excluded). From all foraging trips recorded for each individual we calculated: the mean centroid azimuth (direction of foraging trips, calculated by the centroid point's mean angular difference from the true North for all foraging trips, where more negative values indicate more offshore directions); the maximum ocean depth during a foraging trip averaged across all trips (more negative values indicate greater depths); and the mean distance traveled across days. To summarize the foraging variables for subsequent analyses, we performed a principal component analysis (PCA; Michael et al. 2018). For simplicity in the interpretation of results, since values of azimuth centroid and depth of foraging locations are negatively related to distance traveled, azimuth and depth were multiplied by −1 before the PCA was performed. The first principal component (PC) of the PCA (hereafter, foraging behavior) explained 67.94% of the variance, with load factors of centroid azimuth = 0.63, depth = 0.66, and distance traveled = 0.40. Hence, increasingly positive values of the foraging PC indicate foraging habits of traveling longer distances toward more offshore and deeper waters. Due to our failure to recover the data from a subset of GPS loggers as a result of flooding, internal failure of the instrument, or missing devices, final sample size for the foraging dataset was 39 individuals (16 males and 23 females, from 24 pairs). Data for both male and female partners from the same nest were available for only 15 nests.

### 2.2 Parental Care Effort

During the 10 days of GPS tracking, we performed behavioral observations for all nests (whether parents had GPS or not) from 15:00 to 17:00 h, ending before sunset, a period of high parental care activity (Montoya and Torres 2015). Observations started a day after adults were captured for the first time, and were performed every third day, yielding a total of five behavioral observations per nest. Behavioral observations were performed by five researchers, each one of them observing simultaneously a maximum of four nests, located at a distance of 3–6 m from the researcher. Before the study started, we trained the observers and assessed between‐observers reliability; consistency between‐observers was ≥ 94% before we started the behavioral observations of focal nests. We recorded: (1) the number of begging bouts, when the chicks move their head up and down repetitively toward the male or the female parent, while sometimes making vocalizations; a new begging bout was considered after 10 s of no begging, (2) chick provisioning by the male and the female, when the chicks introduce their beak into one of the parents' beaks for food transfer, and (3) the time when the male or the female parent arrived or departed the nest. To have comparable estimates of parental provisioning effort, one nest was excluded from parental provisioning analyses as only 6 h of observation were recorded. Hence, the number of families for provisioning behavior analyses was 33 (mean total time of observation per nest = 9.74 h, range = 8–10 h). Before the analyses, we calculated the rate of begging (number of beggings/h) to the male or the female (total number of begging bouts toward each parent/total time each parent was present at the nest) and the rate of provisioning (provisioning/h) by the male or the female (total number of provisioning/total observation time) for each nest.

### 2.3 Telomere Length

Using the red blood cells of 66 samples from 33 pairs (blood samples from 1 pair were not collected during fieldwork), we performed DNA extractions using the D4069 Quick‐DNA Plus Kit Miniprep 200 Preps (Zymo Research). All DNA extractions had a proper purity, estimated by the 260/280 ratio (mean = 1.81, range = 1.5–1.93). Immediately after extracting the DNA, we calculated the relative telomere length of individuals by real‐time qPCR following a protocol previously reported for our study species (Guillen‐Parra et al. 2024). Briefly, we amplified both a telomeric (primers: Telomere F1b: CGGTTTGTTTGGGTTTGGGTTTGGGTTTGGGTTTGGGTT and Telomere R2b: GGCTTGCCTTACCCTTACCCTTACCCTTACCCTTACCCT) and a nuclear (single copy gene: Interferon Regulatory Factor 2, IRF2; primers: IRF2 F: GCCTGTGATACTCTCCAGCT and IRF2 R: GGCCCAAGTCTACAAAGTGC) region.

Real‐time qPCRs were performed in a StepOnePlus (Applied Biosystems). Each reaction consisted of a total volume of 25 μL, containing 9.6 ng of template DNA, primers at a final concentration of 500 nM, and 12.5 μL of Luminaris Color HiGreen High ROX qPCR Master Mix (Thermo Scientific). Reactions for the telomere and IRF2 regions were performed on separate plates. The qPCR conditions for the telomeric region were as follows: 10 min at 95°C, followed by 40 cycles of 15 s at 95°C, 34 s at 60°C, and 30 s at 74°C. For IRF2, the qPCR conditions were: 10 min at 95°C, followed by 40 cycles of 15 s at 95°C, 30 s at 58°C, and 30 s at 72°C.

Given logistic limitations, amplifications (for both telomeric and nuclear regions) were performed in two separate batches: 30 samples (15 male and 15 female partners) were analyzed in 2019, and 36 samples (18 male and 18 female partners) were analyzed in 2022. We used one sample as a reference (golden sample) that was included in all plates, in both batches. All samples were included in triplicates, setting male and female partners on the same plate. The quantification cycle (Cq) values were highly repeatable for each amplicon in both batches (Tel, Batch one rICCC = 0.91, *p* < 0.001, Batch two rICCC = 0.92, *p* < 0.001; IRF2, Batch one rICCC = 0.98, *p* < 0.001, Batch two rICCC = 0.99, *p* < 0.001). With the golden sample, we calculated the intra and interplate variation (CV), which was ≤ 1% for both amplicons in both batches. The efficiency of each amplicon was estimated from the amplification curve slopes for each qPCR reaction and was averaged for each gene using the LinRegPCR software. The mean reaction efficiency for telomeres was 1.83 and 1.81 for each batch; and 1.89 and 1.91 for each batch of IRF2.

The relative TL for each sample was calculated from the formula: (Ef Tel) ^ΔCq Tel^/(Ef IRF2) ^ΔCq IRF2^. Where Ef is the amplicon efficiency, and ΔCq is the difference in the Cq values between the golden sample and the focal sample. For the analyses, the mean Cq value obtained from triplicates of each sample was used. To control for potential differences in the telomere length variance between batches, we standardized the relative telomere length within batches using the formula: (Focal value − Mean of the batch)/SD of the batch.

### 2.4 Statistical Analysis

First, to investigate if having a GPS logger for 10 days affects individuals' condition (estimated as the change in body mass during the 10 days of tracking), or their provisioning rate, we performed independent linear mixed models that included whether individuals carried a GPS logger (yes or no), the sex of the parent and the interaction between these two factors as explanatory variables, and nest ID as a random effect. We used a t‐test analysis to compare the daily mass growth rate (estimated as the (ln) final mass measure – (ln) first mass measure/days elapsed between measures) of offspring from parents that were set with a GPS logger and offspring from parents that were not tracked. Since we found no evidence that carrying a GPS logger for 10 days influenced parental condition, provisioning behavior, or chick mass growth rate (see Results), data from parents who carried a GPS logger and those who did not were pooled for subsequent analyses.

We analyzed differences between sexes in foraging behavior (centroid azimuth, mean maximum depth at foraging locations, and mean daily distance traveled), and the foraging behavior index (first principal component, see above) using linear mixed models. Models included the individuals' sex as the explanatory term, and nest ID as a random effect, as in some nests, both pair members were sampled. We tested if the foraging behavior of male and female pair members was correlated using a Pearson's correlation test.

We evaluated the association of parental telomere length with body mass (standardized by sex given the sexual size dimorphism), foraging behavior, and provisioning effort using linear mixed models with normal error distribution that included the nest ID as a random effect. For the first model, body mass was included as the response variable, and telomere length, sex of the parent, and their two‐way interaction as the explanatory terms; ulna length (standardized by sex) was included as a covariate to control for differences in body mass due to variation in size. Models of foraging behavior or provisioning rate as the response variables included the linear and quadratic effects of telomere length of parents, sex of the parent, the interaction telomere length × sex of parent, and as a covariate, the standardized body mass to test if differences in foraging or provisioning are also related to this proxy of individual condition. We tested for quadratic associations of telomere length with foraging and provisioning effort because the relationship between telomere length and breeding performance may not necessarily be linear (Olsson et al. 2011; Bauch, Becker, and Verhulst 2013). The date when GPS tracking started for each individual and the chicks' age did not affect the foraging behavior (Date *χ* ^2^ = 1.72, *p* = 0.19; Chicks' age *χ* ^2^ = 1.32, *p* = 0.25) or provisioning rate (Date *χ* ^2^ = 0.60, *p* = 0.44; Chicks' age *χ* ^2^ = 0.01, *p* = 0.93); hence, we did not include these variables in the analyses to avoid overparameterization. In the model of provisioning rate, the chicks' begging rate was included as a covariate, as this behavior has a positive influence on the parents' provisioning rate (*F*_1,64_ = 12.55, *p* < 0.001). The chick begging rate to the male or the female parent did not differ (*χ* ^2^ = 1.53, *p* = 0.21).

We tested whether differences in foraging and provisioning effort influence the chicks' mass growth rate in separate linear regression models for male and female parents. These models included offspring mass growth rate as the response variable and as explanatory variables the foraging behavior or provisioning rate. Offspring mass growth rate was not affected by their age or sex (chick age *F*_1,30_ = 1.62, *p* = 0.21; chick sex *F*_1,30_ = 0.39, *p* = 0.53), so both variables were excluded from final models to avoid overparameterization.

Finally, since we found that individuals' telomere length was related to foraging behavior, and only for females, foraging behavior predicted the chicks' mass growth rate (see Results), we performed a path analysis to test if the females' telomere length could indirectly affect the chicks' mass growth rate. The path analysis was performed using the *lavaan* package version 0.6–17 in R (Rosseel 2012). First, we compared the “full model,” composed of the whole set of models tested in the previous sections and illustrated in Figure 1 versus the “final model” composed of the two models that evaluated the relationship between the linear and quadratic relationship between the females' telomere length and the foraging behavior and the female foraging behavior and the chicks' mass growth rate. Models were compared using the Akaike Information Criteria (AIC). Finally, only from the “final model,” we estimated the indirect effect of the females' telomere length over the chicks' mass growth rate using the default “Delta method” option (Rosseel 2012).

Statistical analyses were run in R (v 3.5.; 1 R Core Team 2018). Models' statistical assumptions were checked through graphic visualization. Linear mixed models were run using the *lme4* package (Bates et al. 2015). The significance of terms within models was calculated using the “Anova” function in the *car* package (Fox and Weisberg 2019). Nonsignificant interactions (*p* > 0.05) were removed from the final models to have a proper interpretation of the main factors (Engqvist 2005). Throughout the text, mean and standard deviations and standardized estimates of the models are shown.

## 3 Results

During the 10 days of tracking, individuals who were not given a GPS logger and individuals who were tracked did not differ in the change in body mass (GPS *χ* ^2^ = 0.01, *p* = 0.91; Sex *χ* ^2^ = 0.83, *p* = 0.36, GPS * Sex *χ* ^2^ = 0.74, *p* = 0.39), or their chick provisioning rate (GPS *χ* ^2^ = 0.51, *p* = 0.47; Sex *χ* ^2^ = 10.32, *p* < 0.01, GPS * Sex *χ* ^2^ = 0.09, *p* = 0.77). However, independently of whether the birds were tracked or not, females fed their chicks at a higher rate than males (see below). Also, offspring daily mass growth rate was not affected by whether both parents from a nest were tracked with a GPS logger or not (*t*‐test, *t* = −0.07, *p* = 0.94). Therefore, carrying a GPS logger had no measurable impact on the parents or their offspring.

### 3.1 Foraging Behavior

**FIGURE 2:**
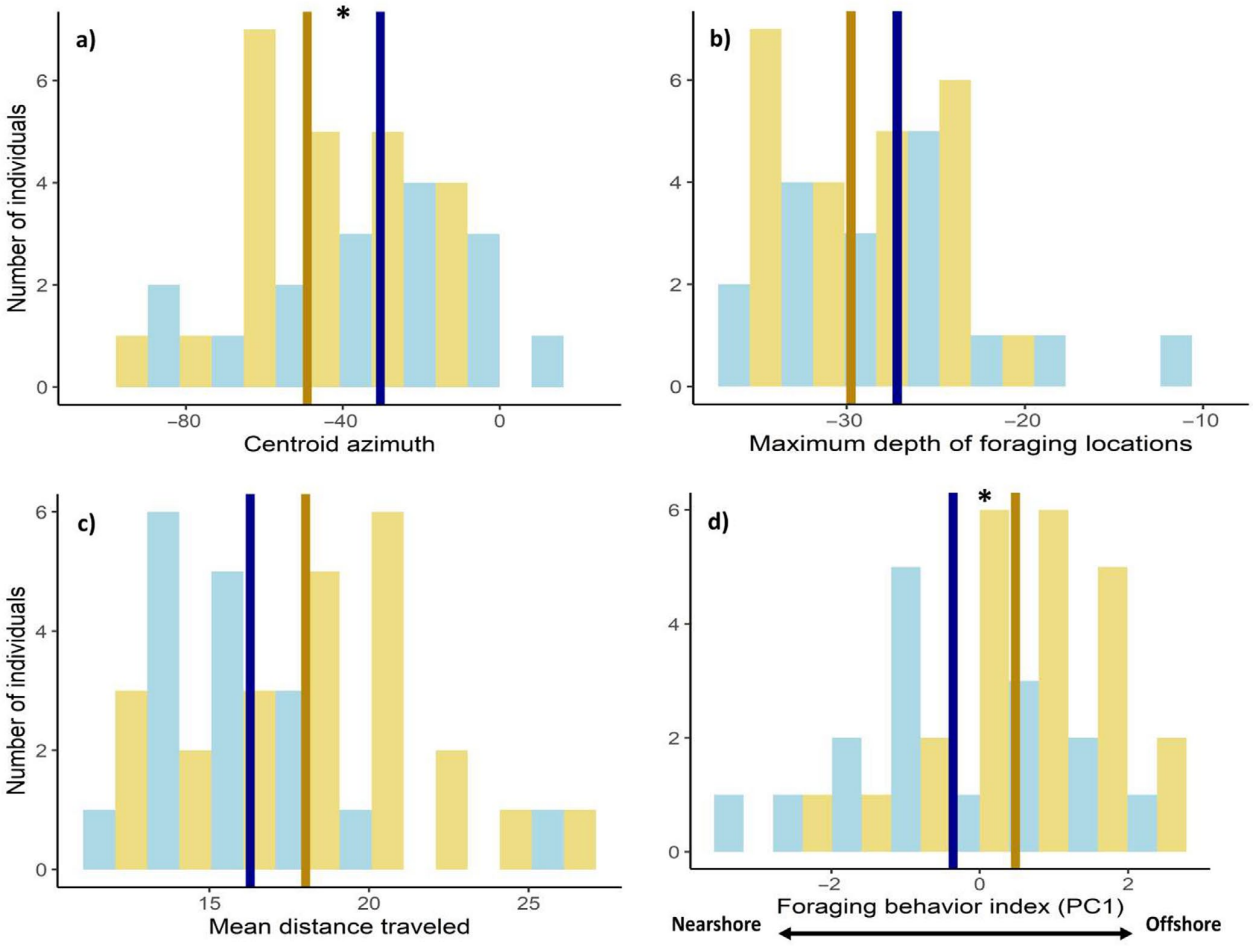
Histograms of males (blue) and females (yellow) variation in foraging parameters. (a) Centroid azimuth. (b) Maximum depth of foraging locations (m). (c) Mean distance traveled (km). (d) Foraging behavior index, obtained from the first principal component of a principal component analysis; increasing values indicate longer foraging trips toward more offshore and deeper waters. Dark blue and gold lines represent, respectively, males' and females' mean for each foraging parameter and asterisks indicate significant differences.

Overall, males and females differed in the direction of their foraging trips relative to the breeding colony (centroid azimuth), with females foraging on average in more offshore locations (−49.11° ± 20.76) than males (−30.43° ± 28.85; Sex *χ* ^2^ = 5.57, *p* = 0.02, Figure 2a). Further, although females tended to cross deeper waters (females: 29.77 ± 4.28 m; males: 27.18 ± 6.05 m; Sex *χ* ^2^ = 2.52, *p* = 0.11, Figure 2b) and travel longer distances than males (females: 18.02 ± 3.70 km; males: 16.28 ± 3.07 km; Sex *χ* ^2^ = 2.49, *p* = 0.11, Figure 2c) the differences were not significant. The three foraging parameters (i.e., centroid azimuth, maximum depth of foraging trips, and mean distance traveled) were correlated (correlation tests, *R* > 0.39, *t* > 2.67, *p* < 0.01); thus, individuals that performed longer foraging trips flew toward more offshore and deeper locations. Our estimated foraging behavior index (PC1 composed of the three foraging parameters, see methods) differed between sexes (Sex *χ* ^2^ = 4.95, *p* = 0.03), suggesting that females exhibited a different foraging syndrome, characterized by a tendency to travel to more offshore and deeper waters at greater distances than males (females: 0.48 ± 1.13; males: −0.36 ± 1.46, Figure 2d). The foraging behavior of male and female partners was not correlated (Pearson's correlation test, *R* = 0.29, *t* = 1.10, *p* = 0.29).

### 3.2 Telomere Length, Body Mass, Foraging Behavior, and Provisioning Rate

Body mass was related to the individuals' telomere length, and this relationship did not differ between sexes (Telomere length *χ* ^2^ = 7.10, *p* < 0.01, Sex *χ* ^2^ = 0.01, *p* = 0.96, Ulna length *χ* ^2^ = 3.50, *p* = 0.06, Telomere length * Sex *χ* ^2^ = 0.91, *p* = 0.34; Model's *r* ^2^ = 0.13). Individuals with longer telomeres had higher body mass (*β* ± SE = 0.34 ± 0.12, *t* = 2.75, *p* < 0.01).

**FIGURE 3:**
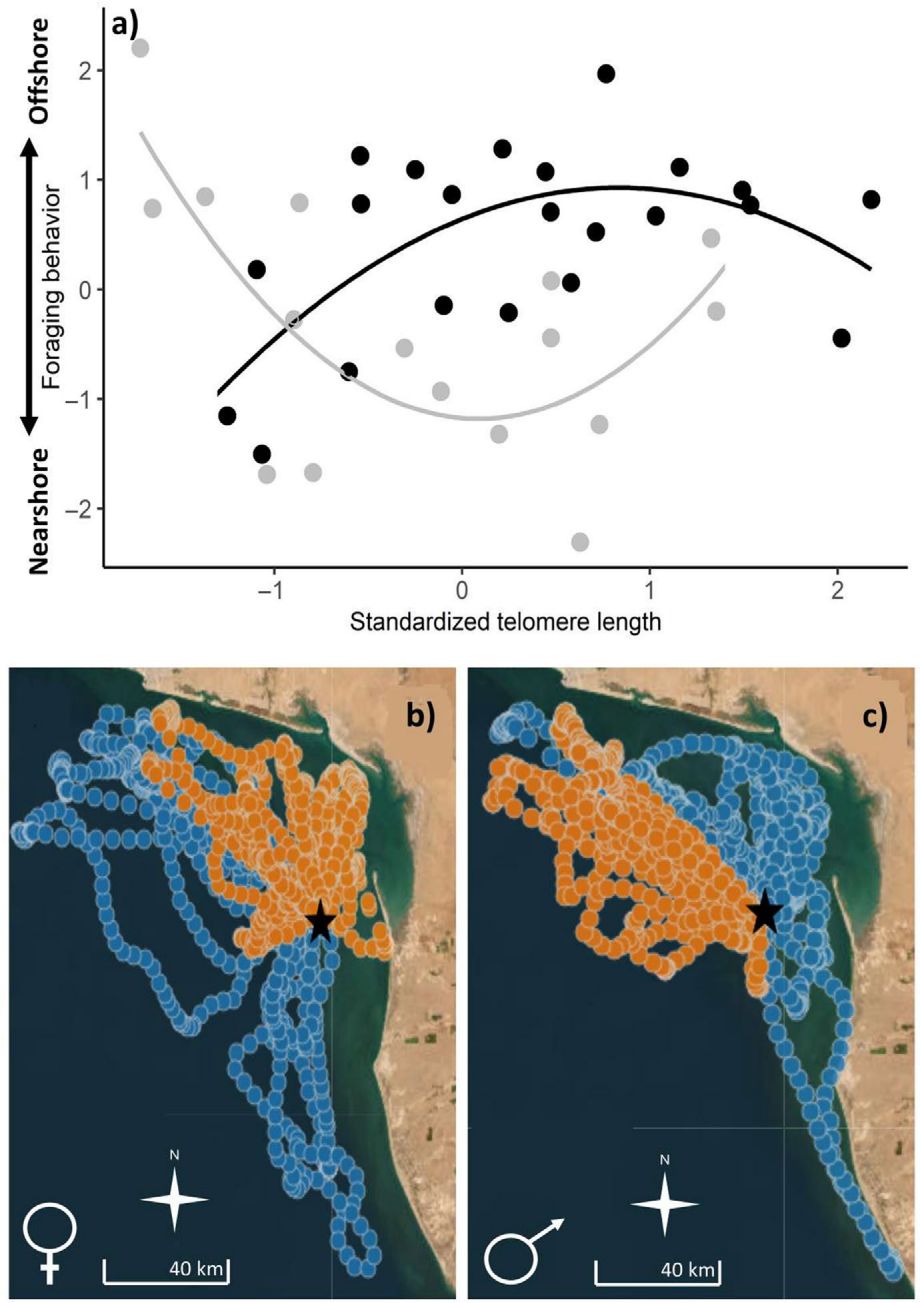
(a) Relationship between the foraging behavior and the standardized telomere length of male (gray circles and line) and female (black circles and line) brown boobies. Foraging behavior was calculated as the first principal component of a principal component analysis where increasing values indicate longer foraging trips toward more offshore and deeper waters. Maps illustrate real foraging behavior showing the continuous locations during all foraging trips registered during the 10 days of GPS tracking of (b) two females and (c) two males with short (orange) and long (blue) telomeres. Black stars indicate the location of the Isla de San Jorge breeding colony. Maps were created using the Tagged Animal Movement Explorer (TAME) software (EcoSHEDS).

Variation in foraging behavior was related to telomere length, but the form of the relationship differed for males and females (Table 1 and Figure 3a); foraging behavior was not related to body mass (Table 1). Females with telomeres of average and longer lengths traveled longer distances toward deeper and offshore areas, compared to females with shorter telomeres (Figure 3a,b). For males, those with shorter telomeres traveled longer distances toward offshore and deeper waters, compared to males with average and longer telomeres (Figure 3a,c).

**TABLE 1:** Results from linear mixed models evaluating the relationship between foraging behavior or chick provisioning rate and parents' telomere length.

	Foraging behavior			Provisioning rate		
	*β* ± SE	*χ* ^*2*^	*p*	*β* ± SE	*χ* ^*2*^	*p*
Telomere length	**0.19 ± 0.18**	**1.11**	**0.29**	**−0.06 ± 0.11**	**0.29**	**0.59**
Telomere length^2^	**−0.18 ± 0.19**	**0.99**	**0.32**	**0.07 ± 0.12**	**0.36**	**0.54**
Sex of parent (Males)	**−0.55 ± 0.27**	**5.37**	**0.02**	**−0.76 ± 0.22**	**11.99**	**< 0.01**
**Body mass**	**−0.08 ± 0.22**	**0.14**	**0.70**	**−0.01 ± 0.05**	**0.04**	**0.85**
Begging rate	—	—	—	**0.35 ± 0.11**	**9.20**	**< 0.01**
Telomere length * Sex of parent	−0.60 **±** 0.30	3.12	0.09	−0.15 **±** 0.25	0.39	0.53
Telomere length^2^ * Sex of parent	**0.98 ± 0.36**	**7.42**	**< 0.01**	−0.11 **±** 0.26	0.19	0.66
Fixed effects marginal *R* ^2^	0.22			0.35		
Random effects, conditional *R* ^2^	0.21			0.01		

*Note:* For the foraging analysis, we used the first principal component from a principal component analysis that included the centroid azimuth, and the mean maximum depth and distance traveled during foraging trips. The sample size was 16 males and 22 females from 23 nests for the foraging analysis and 32 males and 32 females from 32 nests for the provisioning rate analysis. Nest ID was included as a random effect. Initial models included the main terms and the interaction between telomere length and sex; body mass was standardized to control for sexual size dimorphism. Nonsignificant interactions were excluded from final models highlighted in bold.

**FIGURE 4:**
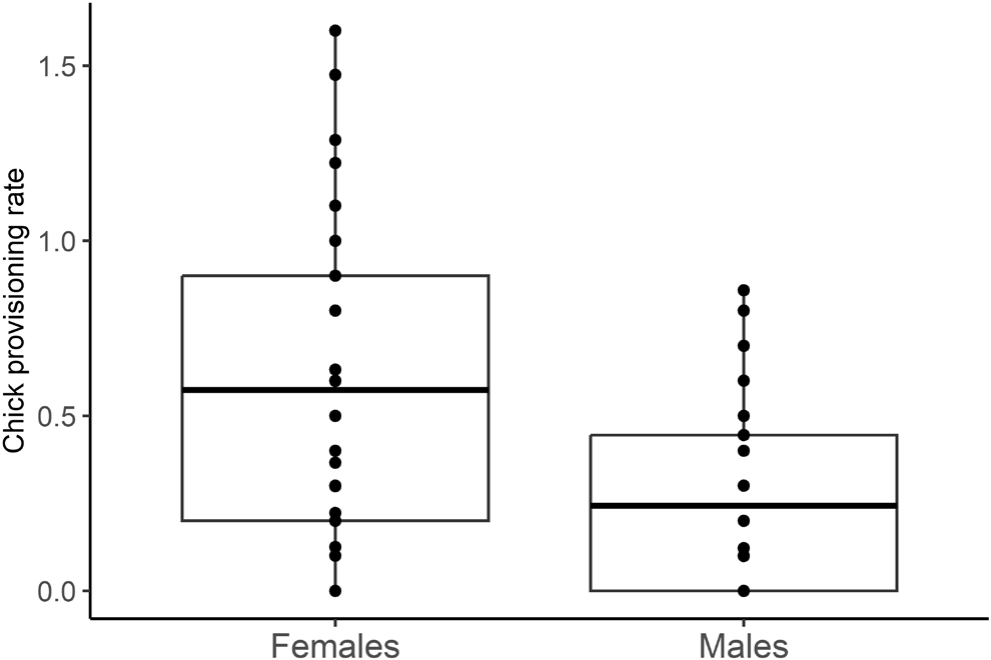
Sex differences in the chick provisioning rate (provisioning/h). In boxplots, the upper and lower lines represent the first and third range distribution quartiles, respectively. Lines inside boxplots show the mean provisioning rate.

The rate of chick provisioning was not related to the parents' telomere length or body mass (Table 1). However, there was a marked sexual difference in provisioning effort: females fed their offspring on average at a rate 2.4 times greater than males (provisioning rate per hour: females 0.57 ± 0.51, males 0.24 ± 0.28; Table 1; Figure 4). The rate of provisioning by parents was positively related to the chicks' begging rate (Table 1).

### 3.3 Offspring Mass Growth Rate and Parental Foraging Behavior and Provisioning Rate

The provisioning rate of males and females was not related to their foraging behavior (Foraging behavior *β* ± SE = −0.20 ± 0.16, *χ* ^2^ = 1.47, *p* = 0.22; Sex (Males) *β* ± SE = −0.58 ± 0.25, *χ* ^2^ = 5.24, *p* = 0.02; Foraging behavior * Sex *β* ± SE = 0.11 ± 0.31, *χ* ^2^ = 0.14, *p* = 0.71).

**FIGURE 5:**
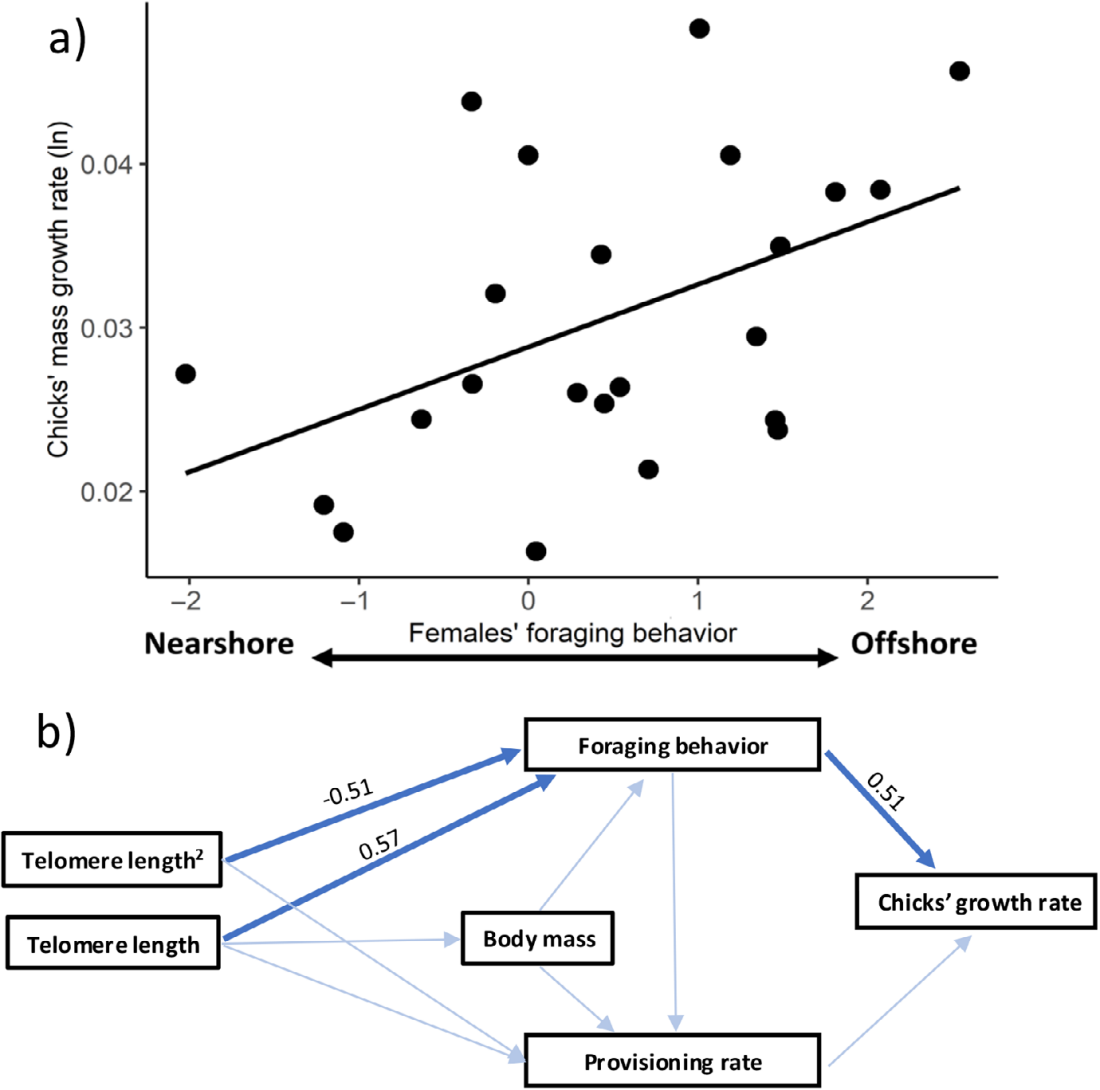
(a) Relationship between the chicks' mass growth rate (natural logarithm transformed) and the females' foraging behavior. Foraging behavior was calculated as the first principal component of a principal component analysis where increasing values indicate longer foraging trips toward more offshore and deeper waters. (b) Path analysis showing the direct paths (blue solid arrows) between the females' telomere length and foraging behavior (linear and quadratic), and the females' foraging behavior and the chicks' mass growth rate. Numbers indicate the standardized estimated effects. Light‐blue arrows represent paths tested in the full model that were not included in the final model.

Chicks' mass growth rate was related to the foraging behavior of the mother (*β* ± SE = 0.55 ± 0.23, *F*_1,21_ = 5.87, *p* = 0.02), but not of the father (*β* ± SE = 0.35 ± 0.25, *F*_1,14_ = 1.98, *p* = 0.18). Females that flew longer distances toward more offshore and deeper waters had chicks with higher mass growth rates (Figure 5a). The chicks' mass growth rate was not explained by the provisioning rate of the mother (*β* ± SE = 0.01 ± 0.17, *F*_1,31_ = 0.01, *p* = 0.97), or the father (*β* ± SE = −0.28 ± 0.16, *F*_1,31_ = 2.97, *p* = 0.10).

The path analysis confirmed that the “final model” was an overall better fit (lower AIC) than the “full model” (AIC, full model = 27.17, final model = −79.27). Females' telomere length had a direct and quadratic effect on foraging behavior (*Z* = −2.12, *p* = 0.03; Figure 5b), and foraging behavior had a positive impact on the chicks' mass growth rate (*Z* = 2.78, *p* < 0.01; Figure 5b). Thus, the results suggest that females' telomere length had a positive indirect effect on the chicks' mass growth rate mediated by its effects on foraging behavior (indirect linear effect, *β* ± SE = 0.29 ± 0.01; Figure 5b).

## 4 Discussion

Brown booby males and females differed in their provisioning and foraging behaviors. Females fed their chicks at higher rates and foraged at more offshore waters than males. Independent of sex, telomere length was positively linked to the parents' body mass, suggesting that individual quality influences the ability of individuals to accumulate body reserves; however, body mass was unrelated to the parents' foraging behavior and provisioning rate. Interestingly, our study underscores a sex‐specific link between foraging behavior and telomere length. Individuals traveled longer distances toward more offshore and deeper waters when having longer or shorter telomeres, depending on whether they were females or males. Only females' foraging behavior influenced chick condition, as chicks had a higher mass growth rate when their mothers foraged in deeper, more offshore locations. Our findings highlight that sex‐specific and interindividual variation in foraging behavior may be linked to differences in individual quality, indicated by telomere length.

Foraging is expected to be constrained by individual quality given the time and energy required, especially while rearing chicks (Shaffer, Costa, and Weimerskirch 2003; Mullers et al. 2009; Lescroël et al. 2020). Accordingly, we found that foraging behavior was related to telomere length, but not to body mass, suggesting that the individuals' intrinsic quality, and not their current condition, explain interindividual variation in foraging. The relationship between telomere length and foraging behavior during chick rearing differed between males and females. Females with longer and average telomere lengths were more prone to forage longer distances toward more offshore and deeper waters, whereas males with longer and average telomere lengths performed more nearshore foraging trips. Interestingly, individuals with shorter telomeres had different foraging behavior compared with same‐sex individuals with longer telomeres. Females with shorter telomeres and presumably lower quality foraged nearshore, possibly due to increased costs of sustaining longer flights to offshore sites. Contrary to our prediction, males with shorter telomeres foraged in deeper, more offshore waters than males with average and longer telomeres, suggesting that the costs and benefits of offshore foraging near Isla de San Jorge may be sex‐dependent. The sample size for males in the present study is small, so results from males should be interpreted with caution. Potentially, offshore foraging in males with shorter telomeres may be a strategy to prioritize self‐maintenance over breeding investment (Saraux et al. 2011), if a diet that includes pelagic prey is of better nutritional quality (Michael et al. 2018). Furthermore, given their presumably poorer quality and lower survival prospects, males with shorter telomeres may be enhancing their parental effort by providing chicks with better food quality through offshore foraging (Giraudeau, Angelier, and Sepp 2019); however, this idea is unlikely in our study as male foraging was unrelated to chick growth. Finally, males and females with shorter telomeres may be excluded from sexually preferred foraging sites through intraspecific competition. Interestingly, the suggestion that males with shorter telomeres foraged at more offshore sites contrasts with findings from a different breeding colony where males performing more offshore foraging trips during courtship and the chick‐rearing period were the ones with greener skin coloration, and presumably better condition (Michael et al. 2018; Michael et al. 2024), yet similar to the present study, it was also found that at Isla de San Jorge during the incubation and chick‐rearing period, males with greener gulars had more benthic (nearshore) diets, suggesting that individuals optimal foraging behavior may depend on both individual quality and environmental heterogeneity (Benowitz‐Fredericks et al. 2022; Michael et al. 2024). Future studies would be needed to untangle the sex‐specific costs and benefits of offshore versus nearshore foraging, specifically considering variation in individual quality and environmental heterogeneity, to evaluate if differences in foraging behavior result from sex‐specific optima.

The chick provisioning rate of mothers and fathers was unrelated to their telomere length, body mass, and foraging behavior, and did not influence the chicks' mass growth rate, a comprehensive indicator of parental investment and success. Importantly, females fed the chicks up to 2.4 times more frequently than males. In theory, sexual differences in parental investment may arise if increased provisioning impacts future reproductive potential of males and females differently (Royle, Hartley, and Parker 2004). Additionally, in species with size dimorphism, sexual differences in food provisioning have been attributed to sexual differences in foraging efficiency or competitive abilities (Lewis et al. 2005; Phillips et al. 2017; Calado et al. 2020). In the brown booby, it is possible that trade‐offs between investing in present versus future reproduction may differ for males and females, and that these differences may be partially linked to sexual size dimorphism. However, we expect these sexual differences to be strongly affected by local environmental conditions as sexual differences in foraging behavior and provisioning rate vary between colonies (Lewis et al. 2005; Montoya and Torres 2015; Castillo‐Guerrero et al. 2016; Miller et al. 2018; Correia et al. 2021). Contrary to our prediction, parental foraging behavior was unrelated to chick provisioning rate, and provisioning rate did not affect the chicks' mass growth rate. Our estimate of provisioning rate cannot ascertain if those chicks that received more feedings per hour received a higher amount of food or food of better quality (see below), which could explain the lack of relationship between provisioning and foraging or chick growth.

We found that the chicks' mass growth rate was affected by the foraging behavior of the mother but not the father. This effect could be indirectly attributed to the mothers' telomere length as suggested by the path analysis. Females that flew longer distances to forage at more offshore and deeper waters, which were also the ones with longer and average telomere lengths, had chicks that increased their mass at higher rates. Brown booby diet in Isla de San Jorge includes both pelagic and benthic fish, as well as squid (Mellink, Domínguez, and Luévano 2001). Interestingly, the diet of brown boobies differs between males and females. Female brown boobies tend to feed on a greater number of species compared to males (Cannell et al. 2022), and rely more on pelagic (offshore) prey while males prefer benthic and demersal (nearshore) prey (Mancini et al. 2023). Furthermore, in the San Jorge colony, the pelagic diet consists mostly of Pacific Anchoveta ( *Cetengraulis mysticetus* , Mellink, Domínguez, and Luévano 2001), which contains high amounts of lipids and calories (González‐Medina et al. 2018). Thus, chicks from parents foraging in offshore locations associated with a more pelagic diet (Michael et al. 2018) may receive a diet richer in caloric content, enabling a faster mass increase. In a cross‐fostering experiment performed on a different colony, provisioning rate did not differ between males and females, and the chicks' body mass gain was affected by both the fostering male and female provisioning rate and by the genetic fathers' condition, which was estimated by their gular color (Montoya and Torres 2015). In contrast, we have no evidence in the present study that male foraging or provisioning rate influences chick mass growth rate, suggesting that environmental heterogeneity in resource abundance between colonies may influence male and female breeding strategies (Escalante et al. 2013). Overall, our results show that females' telomere length is related to their foraging behavior (possibly linked to the quality of diet provided to chicks), which ultimately influences chicks' mass growth rate.

Sexual differences in foraging behavior during breeding occur in many seabird species (Phillips et al. 2017). Although there is no consensus about the causes, the nonexclusive hypotheses to explain these differences in foraging behavior include size dimorphism, habitat specialization, sex‐specific nutrient requirements, and parental task division (Lewis et al. 2005; Phillips et al. 2017; Miller et al. 2018; Calado et al. 2020). Our results suggest that in the brown booby variation between individuals and sexes in the foraging behavior may be partially determined by individual quality, indicated by their telomere length. Interestingly, brown boobies mate assortatively based on telomere length (Guillen‐Parra et al. 2024). This pairing pattern could favor parental tasks division if, as our study suggests, male and female partners of similar telomere length tend to forage in different locations (offshore versus nearshore) resulting in sexual differences in parental contribution to feeding. In the present study, we found no correlation between the foraging behavior of male and female partners. However, the sample size for this analysis was small, so results should be interpreted with caution. Future research could investigate whether telomere length‐dependent sexual differences in foraging behavior may be a cause or consequence of parental task division.

Understanding the causes of sex‐specific variation among individuals in foraging behavior is important for seabird ecology, as this variation is linked to the use of resources and may ultimately underlie the ability of individuals to adapt to environmental changes (Phillips et al. 2017; Calado et al. 2020). Our study provides a proximate link between individual and sexual variation in key activities such as foraging and supports the idea of telomere length as an indicator of the individual's quality and, as an extension, parental quality (Angelier et al. 2019; Viblanc et al. 2020). Furthermore, offspring from females with longer telomeres that foraged offshore showed a greater mass growth rate, indicating that individual quality‐driven variation in foraging behavior may impact reproductive success. The sex‐specific link between telomere length and foraging behavior suggests that individual quality influences the allocation strategy between foraging for self‐maintenance versus parental care, yet males and females may differ in their optimal resolution.

## Author Contributions

**Mauricio Guillen‐Parra:** conceptualization (lead), data curation (lead), formal analysis (lead), investigation (lead), methodology (lead), validation (lead), visualization (lead), writing – original draft (lead), writing – review and editing (lead). **Rocio Barcenas‐Flores:** data curation (supporting), investigation (equal), methodology (equal), writing – review and editing (supporting). **Alberto Velando:** conceptualization (lead), formal analysis (supporting), supervision (equal), writing – review and editing (lead). **Anne Wiley:** conceptualization (equal), funding acquisition (lead), investigation (equal), methodology (equal), writing – review and editing (equal). **Bibiana Montoya:** conceptualization (equal), funding acquisition (lead), investigation (equal), writing – review and editing (equal). **Roxana Torres:** conceptualization (lead), data curation (equal), formal analysis (equal), funding acquisition (lead), investigation (lead), methodology (lead), project administration (lead), supervision (lead), validation (equal), visualization (supporting), writing – review and editing (lead).

## Ethics Statement

All applicable international, national, and institutional guidelines for the care and use of animals were followed. Permissions to conduct the study were granted by Secretaría de Medio Ambiente y Recursos Naturales and Consejo Nacional de Áreas Naturales Protegidas (SGPA/DGVS 04708‐16, 011542‐17). The study was approved by the University of Akron's Institutional Animal Care and Use Committee (Protocol 16‐06‐13‐WBC).

## Conflicts of Interest

The authors declare no conflicts of interest.

## Data Availability

Data are available on Dryad. https://doi.org/10.5061/dryad.51c59zwhf.
